# Diagnostic approach using ERCP‐guided transpapillary forceps biopsy or EUS‐guided fine‐needle aspiration biopsy according to the nature of stricture segment for patients with suspected malignant biliary stricture

**DOI:** 10.1002/cam4.1034

**Published:** 2017-02-21

**Authors:** Yun Nah Lee, Jong Ho Moon, Hyun Jong Choi, Hee Kyung Kim, Seo‐Youn Choi, Moon Han Choi, Tae Hee Lee, Tae Hoon Lee, Sang‐Woo Cha, Sang‐Heum Park

**Affiliations:** ^1^ Department of Internal MedicineDigestive Disease Center and Research InstituteSoonChunHyang University School of MedicineBucheon and SeoulKorea; ^2^ Department of PathologySoonChunHyang University School of MedicineBucheonKorea; ^3^ Department of RadiologySoonChunHyang University School of MedicineBucheonKorea

**Keywords:** Bile duct neoplasm, endoscopic retrograde cholangiopancreatography, endoscopic ultrasound‐guided fine needle aspiration, malignant biliary stricture, pancreatic neoplasm

## Abstract

In malignant biliary stricture (MBS), the diagnostic accuracy of ERCP‐based tissue sampling is insufficient. EUS‐guided fine needle aspiration biopsy (EUS‐FNAB) is emerging as a reliable diagnostic procedure. This study aimed to evaluate the usefulness of a diagnostic approach using ERCP‐guided transpapillary forceps biopsy (TPB) or EUS‐FNAB according to the characteristics of suspected MBS. Consecutive patients diagnosed with suspected MBS with obstructive jaundice and/or cholangitis were enrolled prospectively. ERCP with intraductal ultrasonography (IDUS) and TPB were performed as initial diagnostic procedures. Based on the results of imaging studies and IDUS, all MBS were classified as extrinsic or intrinsic type. If the malignancy was not confirmed by TPB, EUS‐FNAB for extrinsic type or second TPB for intrinsic type was performed. Among a total of 178 patients, intrinsic and extrinsic types were detected in 88 and 90 patients, respectively. The diagnostic accuracy of first TPB was significantly higher in the intrinsic than in the extrinsic type (81.8% vs. 67.8, *P *=* *0.023). In 33 patients with extrinsic type and negative for malignancy on first TPB, the diagnostic accuracy of EUS‐FNAB was 90.9%. In 19 patients with intrinsic type and negative for malignancy on first TPB, the diagnostic accuracy of second TPB was 84.2%. The diagnostic accuracies of the combination of initial TPB with EUS‐FNAB and second TPB were 96.7% and 96.6%, respectively. A diagnostic approach using EUS‐FNAB or TPB according to the origin of MBS is considered effective to improve the diagnostic accuracy of MBS with negative for malignancy on first TPB. (Clinical trial registration number: UMIN000016886).

## Introduction

Endoscopic retrograde cholangiopancreatography (ERCP) is used as a first‐line diagnostic and therapeutic procedure in patients with suspected malignant biliary stricture (MBS). ERCP enables determination of the location and extent of the biliary stricture, and allows tissue samples to be obtained for histopathologic diagnosis. However, the diagnostic yield of ERCP‐based tissue sampling for biliary stricture has been reported to be unsatisfactory and is affected by several factors, such as the location, size and type of stricture, cytology preparation and interpretation, and the skill and experience of the endoscopist 1.

Various endoscopic and radiographic imaging modalities have been studied with the aim of improving the diagnosis of biliary obstruction 2, 3, 4, 5. Among them, endoscopic ultrasonography (EUS) and intraductal ultrasonography (IDUS) have been used increasingly to diagnose and stage the resectability of pancreatobiliary cancers 6. EUS allows tissue sampling for histopathologic diagnosis using fine‐needle aspiration biopsy (FNAB) and exhibits a high diagnostic yield for solid pancreatic masses 7, 8. The sensitivity of EUS‐guided FNAB (EUS‐FNAB) for the diagnosis of indeterminate biliary strictures has been reported to be 43–86% in patients with prior nondiagnostic ERCP 9, 10, 11, 12, 13. However, EUS‐FNAB has several limitations as a routine clinical procedure for all biliary strictures.

Although newer modalities are emerging, the optimal method for the sampling of biliary stricture tissue is unclear. Therefore, a multidisciplinary approach based on the advantages and disadvantages of each modality is required to optimize the diagnosis of suspected MBS. The aim of this study was to evaluate the usefulness of a diagnostic approach using ERCP‐guided transpapillary forceps biopsy (TPB) and EUS‐FNAB according to the nature of the strictured segment in patients with suspected MBS.

## Methods

### Patients

Consecutive patients referred for pathologic confirmation of suspected MBS were prospectively enrolled in this study. The inclusion criteria were (1) the stricture of extrahepatic bile duct due to a thickened bile duct wall or pancreatobiliary mass revealed by cross‐sectional radiologic images (computed tomography [CT], and/or magnetic resonance imaging [MRI]); (2) clinical findings of obstructive jaundice and/or cholangitis; (3) age >18 years; and (4) ability to provide informed consent. The exclusion criteria were (1) underlying pancreatobiliary diseases (e.g., primary biliary cirrhosis or postoperative biliary stricture) without clinical and radiologic image findings suggestive of malignancy; (2) the presence of any contraindication to ERCP; (3) coagulopathy (international normalized ratio *>*1.5 [0.85–1.25] or platelet count <80,000/mm^3^ [150,000–450,000/mm^3^]); (4) difficulty to perform ERCP due to altered gastrointestinal anatomy or a significant duodenal obstruction. Our Institutional Review Board approved this study, and written informed consent was obtained from all enrolled patients. This study was registered in the UMIN Clinical Trial Registry (UMIN000016886).

### Initial diagnostic approach using ERCP with TPB

All procedures were performed using standardized protocols by two experienced investigators using a standard duodenoscope (JF or TJF‐260V; Olympus Medical System Co., Ltd., Tokyo, Japan) with the patients under conscious sedation. Standard techniques were used to cannulate the biliary tract, and contrast (Omnipaque; GE Healthcare, Seoul, Korea) was injected to identify the location and length of the stricture. For all patients, IDUS was performed followed immediately by cholangiogram. The IDUS probe (UM‐G20‐29R; Olympus Medical System) was 2.0 mm diameter and had a radial scanning catheter with a scanning frequency of 20 MHz. The catheter had a monorail‐type design at the tip, which facilitates its passage over guide wires. The IDUS probe was inserted into the bile duct under endoscopic and fluoroscopic guidance over a guide wire without endoscopic sphincterotomy (EST). IDUS imaging was performed during probe withdrawal and stricture dilation was not performed for the IDUS examination. The TPB of the biliary stricture was obtained under fluoroscopically guided retrograde biliary biopsy during ERCP after EST. TPB yielded four to six specimens using forceps with a 1.8‐mm‐diameter cup (FB‐39Q; Olympus Medical System). The remainder of the ERCP procedure was then performed according to the individual needs of each patient.

### Classifications of the type and location of biliary stricture

Biliary stricture was classified as intrinsic or extrinsic type. An extrinsic mass compressing bile duct at the stricture site observed on cross‐sectional images was classified as extrinsic type. Among the remaining patients, an extrinsic mass confirmed by IDUS during ERCP was also classified as extrinsic type. Patients in whom definite extrinsic masses compressing bile duct were not evident on cross‐sectional images and IDUS were classified as intrinsic type. According to the location of stricture, proximal biliary stricture was defined as those located in and/or proximal (relative to the liver hilum) to the upper one‐third of the bile duct. And, mid and lower biliary strictures were defined as those located in the middle one‐third and distal one‐third, respectively.

### Follow‐up biopsy using EUS‐FNAB and TPB according to type of biliary stricture

**Figure 1:**
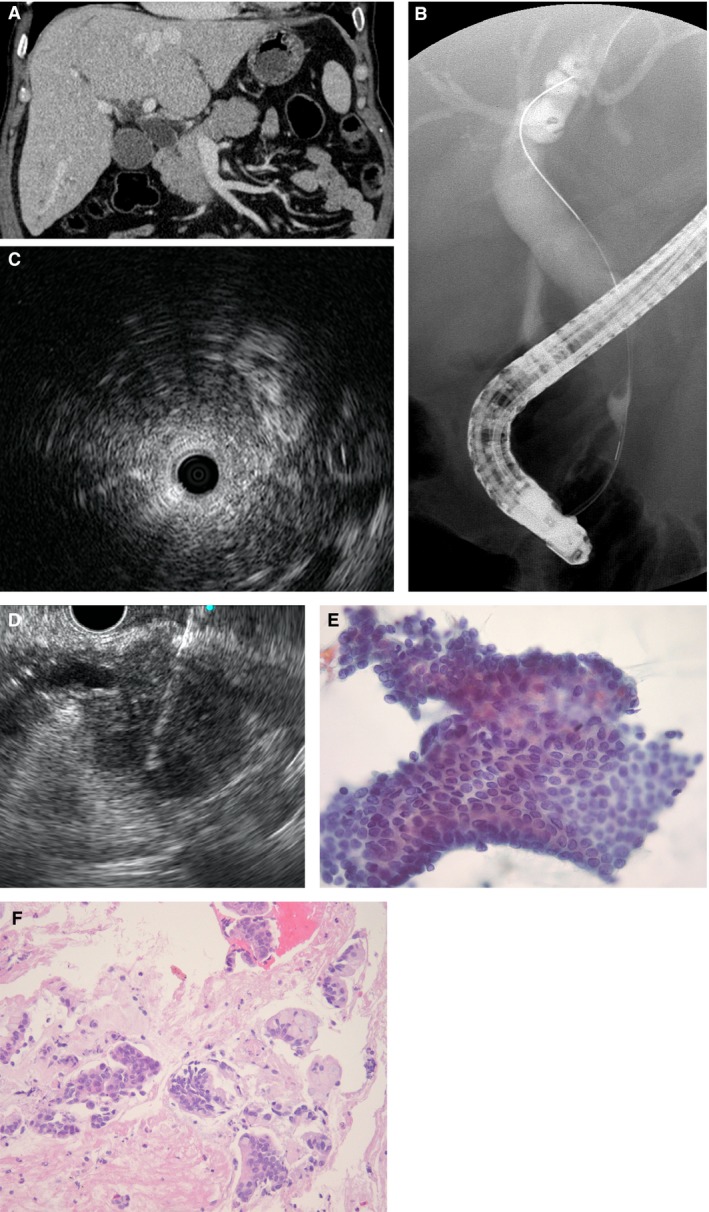
Suspected malignant biliary stricture of the extrinsic type. (A) Computed tomography shows a stricture causing an abrupt and asymmetric narrowing of the common bile duct (CBD) without an extrinsic mass. Cholangiogram showing (B) dilated common hepatic duct with a stricture on the CBD. (C) Intraductal ultrasonography was performed and shows an extrinsic compressing mass of the bile duct. After confirmation of negative for malignancy in initial transpapillary forceps biopsy, (D) EUS‐guided fine‐needle aspiration biopsy was performed as a follow‐up biopsy. Positivity for malignancy was confirmed by (E) cytology (Papanicolaou stain, ×400) and (F) histology (H&E stain, ×400).

In patients with extrinsic type, if malignancy was not detected on the initial TPB for suspected MBS, a follow‐up biopsy was performed by EUS‐FNAB (Fig. 1). EUS‐FNAB was performed in a standardized manner by two experienced investigators using a linear‐array echoendoscope (GF‐UCT240; Olympus Medical Systems) in patients under conscious sedation. FNAB was performed from the stomach using a standard 22 gauge (G), or from the duodenum with a 25G, FNAB device (Echotip ProCore; Wilson‐Cook Medical, Winston‐Salem, NC). After puncturing the lesion, the stylet was removed, and suction was applied using a 5–10 mL syringe. The needle was then moved to and fro 15–20 times and withdrawn from the lesion after suction was released. For specimen preparation and analysis, we used a triple approach comprising simultaneous cytopathologic and histologic evaluations with on‐site examination 14. In patients with intrinsic type, the follow‐up biopsy was performed by a second TPB using the same method as the initial TPB during ERCP.

### Classification of biopsy results

One expert pathologist who was not blinded evaluated the pathologic results of TPB and EUS FNAB. The diagnosis was categorized into nondiagnostic (insufficient tissue sampling for pathologic diagnosis), atypical, negative, and positive for malignancy. Samples considered positive were categorized as positive for malignancy, whereas samples considered nondiagnostic, atypical, and negative were categorized as negative for malignancy. The diagnosis of malignancy on EUS‐FNAB was established when at least one of the specimen preparation methods (on‐site cytology, definite cytology, or histology) was positive for malignancy.

### Standard of reference for final diagnosis

The final diagnosis was made using one of the following methods: (1) definite proof of malignancy in a surgical specimen or biopsy of a metastatic lesion; (2) malignant diagnosis by TPB or EUS‐FNAB and clinical/imaging follow‐up compatible with malignant disease; and (3) no proof of malignancy on TPB and FNAB and during clinical/imaging follow‐up of at least 6 months.

### Outcome measurements

The primary outcome measure was the accuracy of the diagnostic approach using ERCP‐guided TPB and EUS‐FNAB according to the type of biliary stricture in patients with suspected MBS. The secondary outcomes were as follows: the accuracy of initial TPB, technical failure and adverse event rates of ERCP‐guided TPB and EUS‐FNAB. The diagnostic accuracy was defined as the ratio of the sum of true‐positive and true‐negative values divided by the number of lesions, where “true‐positive” referred to the presence of malignant cells. Technical failure was defined as a failure of forceps positioning into the site of the bile duct stricture during TPB or a needle malfunction during EUS‐FNAB. Adverse events were defined as any postprocedure event attributable to the ERCP or EUS‐FNAB. All patients underwent follow‐up investigations with laboratory and radiologic tests for at least 1 day after ERCP or EUS‐FNAB. Excessive bleeding at the site of biopsy, perforation, cholangitis, and pancreatitis were recorded according to normal practice 15.

### Statistical analysis

Categorical parameters including sex, location of stricture, final diagnosis, and adverse event were expressed as frequencies and percentages and compared by *χ* ^2^ test or Fisher's exact test. Continuous variables including age, length of stricture, and number of samplings during first TPB were summarized as means ± standard deviation (SD) and were compared using an unpaired *t*‐test. Sensitivity, specificity, and accuracy analyses were performed, and the data are reported with exact 95% confidence intervals (CIs) and compared with the Fisher's exact test for computing *P* values. All statistical analyses were performed using the SPSS for Windows software package (ver.19.0; SPSS Inc., Chicago, IL), with a *P* < 0.05 taken to indicate statistical significance. All authors had access to the study data and reviewed and approved the final manuscript.

## Results

**Figure 2:**
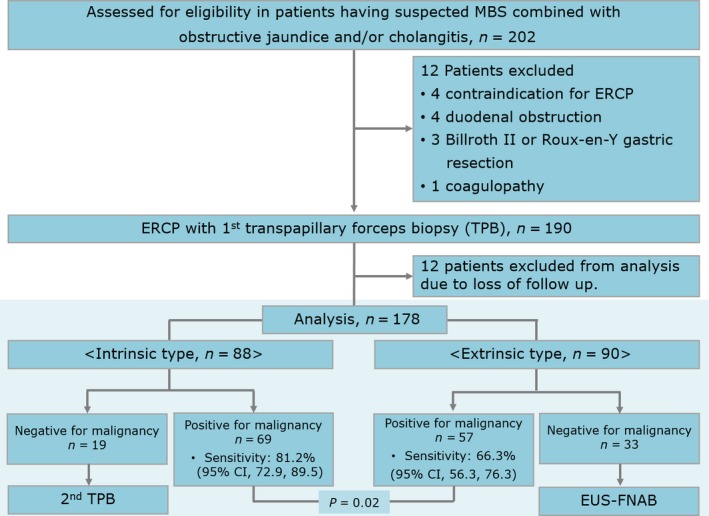
Patient flow chart. MBS, malignant biliary stricture; EUS‐FNAB, endoscopic ultrasonography‐guided fine‐needle aspiration biopsy; CI, confidence interval.

Between January 2012 and March 2014, 202 patients were screened; of these, 12 were excluded for the following reasons: contraindication to ERCP (*n *=* *4), inability to perform an ERCP due to duodenal obstruction (*n *=* *4), previous Billroth II or Roux‐en‐Y gastric resection (*n *=* *3), and coagulopathy (*n *=* *1). The remaining 190 patients underwent ERCP with first TPB, but 12 patients were excluded from the analysis because the final diagnosis was not confirmed due to follow‐up loss. Finally, a total of 178 patients with suspected MBS were analyzed (Fig. 2). No technical failures occurred during TPB and EUS‐FNAB.

### Classification of the type of biliary stricture

The extrinsic and intrinsic types of biliary stricture were detected in 90 (50.6%) and 88 (48.4%) patients, respectively. Among the patients with extrinsic type, an extrinsic mass not observed by CT and/or MRI was diagnosed by IDUS in four patients (4.4%). IDUS probe could pass through the stricture in 176 patients (98.9%), except two patients diagnosed extrinsic mass by previous CT. The demographic characteristics and stricture lengths were not significantly different between the intrinsic and extrinsic types. The most frequent stricture location was the proximal common bile duct (CBD) (55.7%) for intrinsic type and the distal CBD (61.1%) for extrinsic type (*P *<* *0.001). The final diagnosis of suspected MBS in the extrinsic type was pancreatic cancer (*n *=* *64), gallbladder cancer (*n *=* *13), hepatocellular carcinoma (*n *=* *6), metastatic cancer (*n *=* *3), and benign (*n *=* *4). The final diagnosis of the intrinsic type was cholangiocarcinoma (*n *=* *84), metastatic cancer (*n *=* *1), and benign (*n = *3) (Table 1).

**Table 1:** Baseline characteristics and final diagnoses of patients with suspected MBS

	Type of suspected MBS		*P*
	Intrinsic (*n *=* *88)	Extrinsic (*n *=* *90)	
Age, years	71 (11)	68 (12)	0.099
Sex (male/female), *n*	52/36	51/39	0.152
Length of stricture, mm	30 (11)	27 (10)	0.147
Location of stricture, *n* (%)			<0.001
Proximal	49 (55.7)	12 (13.3)	
Mid	25 (28.4)	23 (25.6)	
Distal	14 (15.9)	55 (61.1)	
Number of samplings during first TPB	4.3 (0.6)	4.2 (0.5)	0.152
Final diagnosis, *n* (%)			0.513
Malignant	85 (96.6)	86 (95.6)	
Cholangiocarcinoma	84	0	
Pancreatic cancer	0	64	
Gallbladder cancer	0	13	
Hepatocellular carcinoma	0	6	
Metastatic cancer	1	3	
Benign	3 (3.4)	4 (4.4)	

Quantitative data are expressed as means (±standard deviation).

MBS, malignant biliary stricture; TPB, transpapillary forceps biopsy.

### First TPB during ERCP

The overall diagnostic accuracy of first TPB in a total of 178 patients was 74.7% (95% CI: 68.3, 81.1). The first TPB during ERCP showed positivity for malignancy in 69 patients with the intrinsic type and 57 patients with the extrinsic type. The diagnostic accuracy of first TPB was significantly higher in patients with the intrinsic type than those with the extrinsic type (81.8% vs. 67.8, *P *=* *0.023) (Table 2). Adverse event‐related TPB was not observed.

**Table 2:** Pathologic results and diagnostic accuracies of first transpapillary forceps biopsy in patients with suspected MBS

Pathologic result, *n*	Total (*n = *178*)*	Type of suspected MBS		*P*
		Extrinsic (*n *=* *90)	Intrinsic (*n *=* *88)	
Malignant	126	57	69	
Atypia	40	24	16	
Benign	9	7	2	
Nondiagnostic	3	2	1	
Accuracy, % (95% CI)	74.7 (68.3, 81.1)	67.8 (56.3, 76.3)	81.8 (73.8, 89.9)	0.023^a^

MBS, malignant biliary stricture; CI, confidence interval.

a Comparison of the intrinsic and extrinsic types.

### Follow‐up biopsy using EUS‐FNAB and second TPB

Thirty‐three patients with the extrinsic type and negative for malignancy on first TPB underwent EUS‐FNAB as a follow‐up biopsy for suspected MBS. Of these, 26 patients (78.8%) were found to be positive for malignancy by EUS‐FNAB. In the intrinsic type, a second TPB was performed as a follow‐up biopsy for 19 patients with negative for malignancy on first TPB. Of these, 13 patients (68.4%) were found to be positive for malignancy by a second TPB. The diagnostic accuracy of EUS‐FNAB in the extrinsic type and second TPB in the intrinsic type was 90.9% and 84.2%, respectively; the difference was not significant (*P *=* *0.823) (Table 3). After TPB and EUS‐FNAB, three cases of mild pancreatitis were observed in one patient (5.3%) with the intrinsic type and two patients (6.1%) with the extrinsic type, respectively (*P *=* *0.701).

**Table 3:** Pathologic results and diagnostic accuracies of follow‐up biopsy of suspected MBS in patients with negative for malignancy on first transpapillary forceps biopsy

Pathologic results, *n*	Total (*n = *52)	Follow‐up biopsy method		*P*
		EUS‐FNAB in extrinsic type (*n *=* *33)	Second TPB in intrinsic type (*n *=* *19)	
Malignant	39	26	13	
Atypia	9	5	4	
Benign	2	1	1	
Nondiagnostic	2	1	1	
Accuracy, % (95% CI)	88.5 (79.8, 97.2)	90.9 (81.1, 100)	84.2 (67.8, 100)	0.823

MBS, malignant biliary stricture; TPB, transpapillary forceps biopsy; EUS‐FNAB, endoscopic ultrasonography‐guided fine‐needle aspiration biopsy; CI, confidence interval.

### Overall diagnostic yield of first TPB combined with follow‐up biopsy

For the extrinsic type, the combination of first TPB and follow‐up EUS‐FNAB for patients with negative for malignancy on first TPB showed a sensitivity of 96.5% for diagnosis of malignancy. For the intrinsic type, the combination of first TPB and follow‐up by second TPB achieved a sensitivity of 96.5%; the difference was not significant (*P *=* *0.653). The overall diagnostic accuracies of a combination of initial TPB with follow‐up biopsy using EUS‐FNAB in extrinsic type, and a second TPB in intrinsic type, were 96.7% and 96.6%, respectively (*P *=* *0.648) (Table 4).

**Table 4:** Overall diagnostic yield of first transpapillary forceps biopsy combined with follow‐up biopsy methods according to type of suspected MBS

	Type of suspected MBS, % (95% CI)		*P*
	Extrinsic^a^ (*n *=* *90)	Intrinsic^b^ (*n *=* *88)	
Sensitivity	96.5 (90.2, 98.8)	96.5 (90.1, 98.8)	0.653
Specificity	100 (51.0, 100)	100 (43.9, 100)	NA
Accuracy	96.7 (93.0, 100)	96.6 (92.8, 100)	0.648

MBS, malignant biliary stricture; CI, confidence interval; NA, not available; TPB, transpapillary forceps biopsy; EUS‐FNAB, endoscopic ultrasonography‐guided fine‐needle aspiration biopsy.

a Based on the combination of first TPB and EUS‐FNAB in patients with negative for malignancy on first TPB.

b Based on the combination of first TPB and second TPB in patients with negative for malignancy on first TPB.

## Discussion

ERCP is the most widely used diagnostic procedure for evaluating patients with bile duct obstruction. Traditionally, it has been used to acquire tissue samples for pathologic diagnosis and to determine the location and extent of the bile duct stricture. ERCP‐based tissue sampling can be achieved by brush cytology or TPB. It is routinely performed to diagnose bile duct stricture because it is technically easy, requires little time, and is generally safe. However, the sensitivity of brush cytology alone for diagnosis of MBS is unsatisfactory (33–58%) 1, 9, 16, 17. In most cases, forceps biopsies had higher yields than brush cytology and percutaneous biopsy. However, the sensitivity of TPB for the diagnosis of MBS is insufficient, ranging from 33% to 89%. In addition, the diagnostic yield of ERCP‐based tissue sampling, including TPB, differed according to the nature of MBS 1, 17, 18, 19. Several factors limit the ability of TPB to enhance the diagnostic yield for subjects with suspected MBS. Tissue sampling during TPB is performed in a somewhat blind manner under fluoroscopic guidance, such that precise targeting of the lesion is problematic. In addition, the diagnosis of MBS using TPB is particularly difficult in patients with the pure extrinsic type, because only the surface of the bile duct mucosa can be acquired by TPB. Moreover, the desmoplastic nature of cholangiocarcinoma, and the relatively low cellularity and firmness of pancreatic cancer, make sampling difficult.

To compensate for the disadvantages of TPB for diagnosis of suspected MBS, several diagnostic modalities have been assessed. Among them, EUS‐guided fine‐needle aspiration (EUS‐FNA) is increasingly being used in the diagnostic evaluation of patients with biliary stricture. EUS‐FNA has become a standard procedure for pathologic diagnosis of pancreatic solid cancer based on the high diagnostic yields reported by previous studies 7, 8, 20. In cases of indeterminate biliary stricture, the sensitivity of EUS‐FNA has been reported to vary from 43% to 86% in patients with prior nondiagnostic ERCP 9, 10, 11, 12, 13. In particular, the sensitivity of EUS‐FNA for proximal bile duct stricture was significantly lower than that for distal bile duct stricture 13, 21. In general, EUS‐FNA is more practical for mass‐forming lesions, such as pancreatic cancer, which is the main cause of distal MBS. However, extrahepatic cholangiocarcinoma is most frequently of the periductal infiltrative type, in which tumor growth progresses along the bile duct longitudinally 22, 23. Therefore, extrahepatic cholangiocarcinoma of the diffuse wall‐thickening type is difficult to diagnose by FNA 10. Furthermore, visualizing and sampling using EUS‐FNA in patients with proximal bile duct lesions is more difficult than in those with distal bile duct lesions because proximal bile duct lesions are further from the tip of the echoendoscope and closer to the liver parenchyma than those in the distal bile duct 6. Weilert et al. 24 reported that EUS‐FNA was more sensitive and accurate than ERCP‐guided tissue sampling methods in 51 patients with suspected MBS (94% vs. 50%, *P *<* *0.0001). However, in that study, a relatively large number of patients with pancreatic cancer (36 of 51 patients [70.6%]) were enrolled. Therefore, the superiority of EUS‐FNA for diagnosis of suspected MBS reported by Weilert et al. seems to be inevitable.

Before it is performed in patients with suspected MBS, several aspects of EUS‐FNA should be considered, in addition to the location of lesion. To perform EUS‐FNA, the endoscope must be exchanged, which is costly and time consuming. Moreover, the success of the technique in terms of sample acquisition and diagnosis is dependent largely on the availability of on‐site cytopathologists 25. Additionally, seeding of the FNA tract by malignant cells has been reported, especially in patients with proximal and mid‐CBD strictures 26, 27, 28, 29. Despite the lack of conclusive data, the risk of needle‐tract seeding during EUS‐FNA should be considered in patients with proximal and mid‐CBD strictures. These concerns have led to EUS‐FNA being considered a contraindication to liver transplant for cholangiocarcinoma at some transplant centers, such as the Mayo Clinic 30, 31.

EUS‐FNAB is an acceptable diagnostic procedure for patients with suspected MBS; however, its use as a first‐line diagnostic procedure for all patients is controversial. TPB and EUS‐FNAB are more adaptable to diagnosis of the intrinsic and extrinsic types of stricture, respectively. Therefore, TPB and EUS‐FNAB are complementary, not alternative, procedures for achieving a confirmative diagnosis in cases of suspected MBS. In this study, we developed a diagnostic approach for patients with suspected MBS and obstructive jaundice and/or cholangitis using TPB and EUS‐FNAB. ERCP with TPB was performed as an initial tissue‐sampling method. If patients with extrinsic and intrinsic types were negative for malignancy by first TPB, EUS‐FNAB and repetitive TPB, respectively, were performed as follow‐up methods. And, this diagnostic approach using a combination of initial TPB with follow‐up biopsy using EUS‐FNAB in extrinsic type, and a second TPB in intrinsic type, showed an acceptable diagnostic accuracy with 96.7% and 96.6%, respectively (*P *=* *0.648). Based on our results, performing EUS‐FNAB after negative for malignancy on initial TPB is acceptable to patients with the extrinsic type in need of a therapeutic ERCP for biliary drainage.

Our study had several limitations. First, it lacked a control group. No direct comparison of TPB and EUS‐FNAB was performed, because this was a descriptive study demonstrating the feasibility and diagnostic yield of a diagnostic approach using TPB and EUS‐FNAB according to stricture type in patients with suspected MBS. Second, all procedures were conducted by two highly experienced endoscopists at a single center, such that the results may not reflect practices or technologies used at other institutions. Third, we did not perform a crossover analysis of EUS‐FNAB and TPB in patients with negative for malignancy.

In conclusion, TPB during ERCP appears still to be a useful initial tool to diagnose in patients with a suspected MBS who develop jaundice and/or cholangitis. In addition, a diagnostic approach using EUS‐FNAB or second endoscopic TPB according to the origin of MBS is considered highly effective to improve the accuracy of histologic diagnosis of MBS in patients with negative for malignancy by a first endoscopic TPB.

## Conflicts of Interest

The authors disclose no conflicts of interest.
